# Regional differences in prognostic value of cardiac valve plane displacement in systemic light-chain amyloidosis

**DOI:** 10.1186/s12968-017-0402-2

**Published:** 2017-11-09

**Authors:** Marco M. Ochs, Thomas Fritz, Nisha Arenja, Johannes Riffel, Florian Andre, Derliz Mereles, Fabian aus dem Siepen, Ute Hegenbart, Stefan Schönland, Hugo A. Katus, Matthias G. W. Friedrich, Sebastian J. Buss

**Affiliations:** 1 Department of Cardiology, University of Heidelberg, INF 410, 69120 Heidelberg, Germany; 2 Department of Hematooncology, University of Heidelberg, INF 410, 69120 Heidelberg, Germany

**Keywords:** Cardiac valve plane excursion, Anterior aortic plane systolic excursion, AAPSE, MAPSE, TAPSE, AL amyloidosis, Risk assessment

## Abstract

**Background:**

To compare the prognostic value of cardiac valve plane displacement (CVPD) on various locations in cardiac light chain (AL) amyloidosis.

**Methods:**

Consecutive patients with biopsy-proven cardiac involvement in AL amyloidosis who had undergone cardiovascular magnetic resonance (CMR) between 2005 and 2014 in our institution, were retrospectively identified and data analyzed. The primary combined endpoint was all-cause mortality or heart transplantation. Systolic CVPD were obtained from standard cine bSSFP in 2-, 3- and 4-chamber views at anterior aortic plane systolic excursion (AAPSE); anterior, anterolateral, inferolateral, inferior, inferoseptal mitral (MAPSE); and lateral tricuspid (TAPSE) annular segments.

**Results:**

We identified 68 patients (58 ± 10 years; 59% male). Median follow-up period was 1.2 years (IQR, 0.3-4.1). Significant differences in CVPD between patients who reached a primary endpoint (*n* = 44) and transplant-free survivors were found only for AAPSE (6.1 mm (IQR, 4.6-9.4) vs. 8.8 mm (IQR, 6.9-10.4); *p* = 0.02) and MAPSE_anterolateral_ (7.3 mm (IQR, 5.4-11.7) vs. 10.5 mm (IQR, 8.1-13.4); *p* = 0.03). AAPSE (χ^2^ = 15.6; *p* = 0.0002) provided the best predictive value for transplant-free survival compared to all other valvular plane locations. A high-risk cutoff (AAPSE ≤ 7.6 mm) was calculated by ROC analysis to predict all-cause death or heart transplantation within 6 months from index examination (AUC = 0.80; CI: 0.68 to 0.89; *p* < 0.0001). AAPSE added incremental prognostic power to an imaging prediction model of late gadolinium enhancement and global longitudinal strain (GLS) (∆χ^2^ = 5.8, p = 0.02) as well as to a clinical model including Karnofsky index and NT-proBNP (∆χ^2^ = 6.2, *p* = 0.01).

**Conclusion:**

In patients with cardiac involvement in AL amyloidosis, systolic CVPD obtained from standard long axis cine views appear to indicate outcome better, when obtained in the anterior aortic plane (AAPSE) and provide incremental prognostic value to LGE and strain measurements.

## Background

The early detection of myocardial protein infiltration is crucial to patient care in patients with suspected cardiac amyloidosis in systemic light chain amyloidosis (AL), as cardiac impairment determines overall prognosis and is present in up to 90% of patients during the course of the disease [1–4]. The median survival of affected patients with markedly elevated brain natriuretic peptide (BNP) and cardiac troponin levels is reduced to just about 8 months [5]. As renal insufficiency is common in AL and levels of serum biomarkers depend on glomerular filtration rate, imaging parameters are of special value in staging the disease.

Longitudinal systolic deformation of the left ventricle (LV) is known to be strongly associated with survival in AL amyloidosis [6–8]. Atrioventricular plane displacement contributes approximately 60% to the LV stroke volume [9]. The typical pattern of cardiac impairment follows a regional gradient from base to apex and is different in other causes of LV hypertrophy [10, 11]. Despite modern and more refined imaging technics, Mitral Annular Plane Systolic Excursion (MAPSE) has remained a widely accepted conventional parameter for rapid assessment of LV longitudinal function. MAPSE was introduced in the late 1980s [12] and was later applied for the evaluation of prognosis in heart failure patients [13]. However, recent findings have suggested an incremental prognostic value of Anterior Aortic Plane Systolic Excursion (AAPSE) by means of M-Mode echocardiography [14]. Systolic excursion patterns are heterogeneous [15] due to the differing passive regional resistance of the four interconnected fibrous rings and the active clockwise helical contraction of attached longitudinal myocardial fibers. Therefore, their predictive power of annular locations may also vary.

The purpose of the present study was a head-to-head comparison of the prognostic value of cardiovascular magnetic resonance (CMR) measurement locations for cardiac valve plane displacement (CVPD) in patients with AL amyloidosis.

## Methods

### Study population and design

**Fig. 1:**
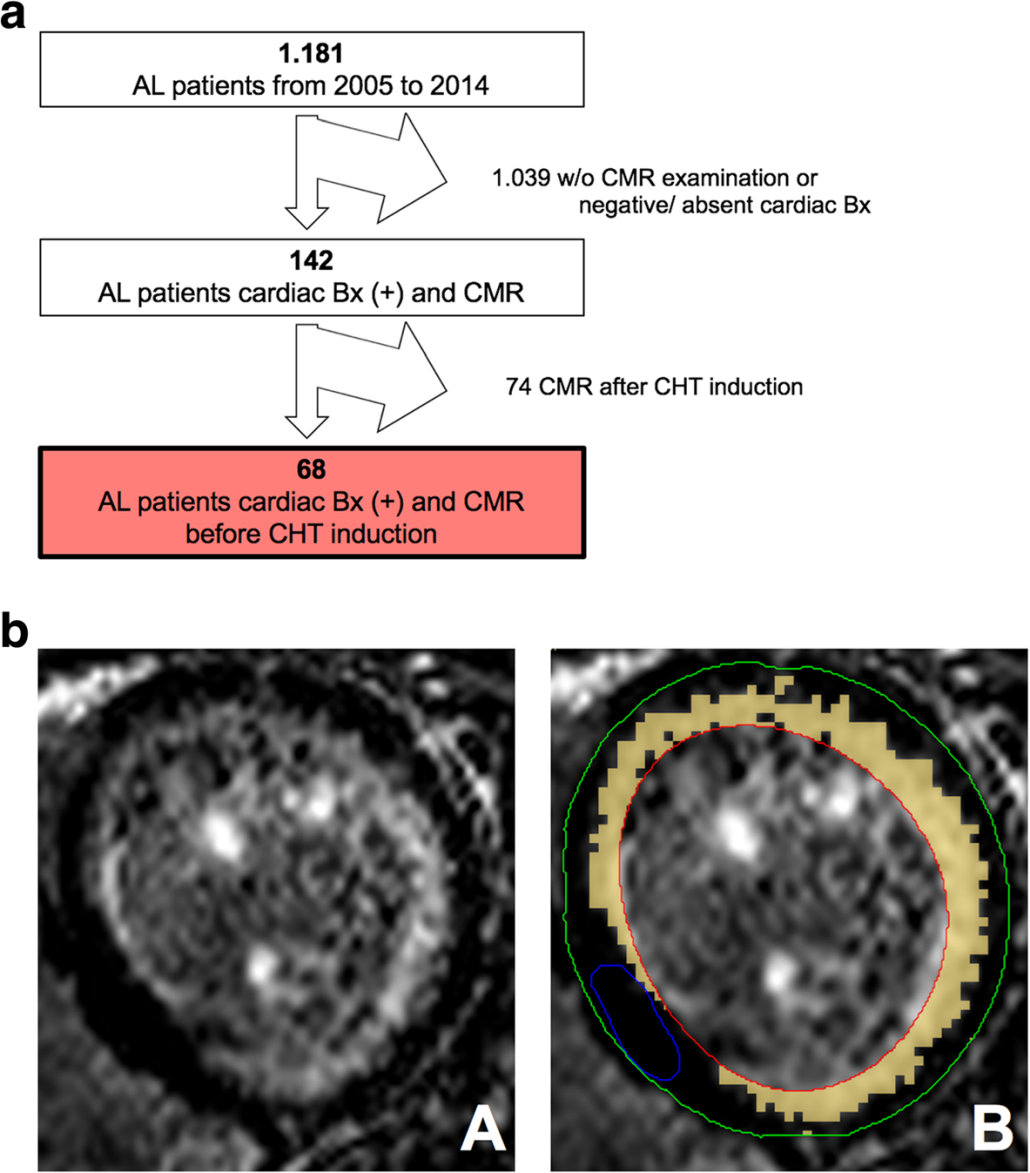
**a** Patient selection flow chart. From 2005 to 2014 a total of 1.181 AL patients visited our institution. Patients had to be excluded for negative or absent cardiac biopsy (Bx), absent CMR examination, or CMR performed after induction of chemotherapy (CHT). The final study cohort consists of 68 AL patients. **b** Late gadolinium enhancement (LGE) quantification by semiautomatic 5 SD-threshold selection. Contours for endo- (red) and epicardial borders (green) as well as for unenhanced reference myocardium (blue) were drawn manually. Signal intensities above 5-fold standard deviation of the reference myocardium were accounted to the LGE volume in relation to unenhanced myocardium (relative LGE, orange)

We identified patients with cardiac biopsy-secured systemic AL amyloidosis who had undergone a CMR between February 2005 and October 2014 at our institution for a retrospective single-center post-hoc analysis. Patient selection is demonstrated in a flow chart (Fig. 1a). The mean interval between CMR and the start of chemotherapy was 23 days. The diagnosis was defined by the presence of monoclonal gammopathy by serum electrophoresis, immunofixation on serum and urine, and free light chain test, and confirmed by positive Congo red staining with birefringence under polarized light of any biopsy (periumbilical fat aspiration, rectum or target organ), positive immunohistology for kappa or lambda in the biopsy, and on the exclusion of hereditary forms of amyloidosis. The study population was referred to our imaging center for cardiologic work up with diagnosed light chain disorder. All 68 patients have signed a written informed consent for the retrospective analysis of clinical routine data. The use of de-identified patient data for research purposes was approved by the institutions ethics committee in accordance to the Declaration of Helsinki.

### CMR acquisition protocol and analysis of parameters

All CMR examinations were performed on a 1.5 Tesla scanner (Achieva, Philips Healthcare, Best, The Netherlands) equipped with a cardiac phased array 32-channel receiver coil. Cine images were obtained using a breath-hold segmented-k-space balanced fast-field echo sequence (bSSFP) employing retrospective electrocardiogram (ECG)- or pulse oximetric gating with 35 phases per cardiac cycle. Typical CMR imaging parameters were: field-of-view (FOV) = 350 × 350 mm^2^, repetition time/echo time (TR/TE) = 2.8/1.4 ms, acquired voxel size = 2.2 × 2.2 × 8 mm^3^, flip angle (FA) = 60°, reconstructed voxel size = 1.3 × 1.2 × 8 mm^3^. Data were analyzed by a single examiner blinded to the patients’ clinical status. Routine analysis was performed on a commercially available workstation (IntelliSpace Portal (ISP) Version 7.0.1, Philips Healthcare, Best, The Netherlands) with a semi-automatic software for volumetric analysis. Ventricular volumes, ejection fraction and LV myocardial mass were acquired in short axis stacks by manually tracing epi- and endocardial borders, excluding papillary muscles from the myocardium. Relative LGE mass was measured using a 5-standard-deviation (SD) threshold in reference to visually unenhanced myocardium (Fig. 1b) [16]. LGE was graduated into (1) none, (2) subendocardial or (3) transmural involvement (qualitative LGE). Global longitudinal strain (GLS) derived from cine SSFP of 2-, 3-, and 4-chamber long axis views by delineating endo- and epicardial borders at end-diastole using CVI cmr^42^ version 5.6.1, (Circle Cardiovascular Imaging, Calgary, Canada). Regional longitudinal strains are presented for basal (mean longitudinal strain of American Heart Association segments 1 to 6), midventricular (mean longitudinal strain of segments 7 to 12) and apical slices (mean longitudinal strain of segments 13 to 16).

### Assessment of cardiac valve plane displacement

Regional systolic valve plane excursion was assessed at seven locations on anterior aortic plane systolic excursion (AAPSE), anterior, anterolateral, inferolateral, inferior and anterolateral mitral (MAPSE), and lateral tricuspid annulus (TAPSE) derived from cine bSSFP images in 2-, 3-, and 4-chamber views as previously described [15]. CVPD was defined as the distance from peak end-diastolic to peak end-systolic excursion of each annular region, where the endocardial insertion points of the aortic, mitral or tricuspid valve leaflets respectively served as anatomic landmarks.

### Blood samples

Blood samples were drawn up to 14 days before the date of CMR examination. NT-proBNP (N-terminal prohormone of brain natriuretic peptide) and cTNT (cardiac troponin T) were obtained from a commercially available fully automated analyzer based on a sandwich immunoassay (ELECSYS, Roche Diagnostics, Mannheim, Germany). Glomerular filtration rate was estimated by the “Modified Diet in Renal Diseases” (MDRD) formula.

### Follow-up

Primary combined endpoint was all-cause mortality or heart transplantation. Follow-up was obtained by review of patient’s electronic records or telephone interview with the patient or relative.

### Statistical methods

Data were analyzed using MedCalc, version 15.11.4 (MedCalc Software, Ostend, Belgium). Continuous and normal distributed variables (Kolmogorov-Smirnov test, *p* ≥ 0.05) were expressed as mean ± standard deviation. Group differences for continuous variables were tested using the independent t-test, and differences between nominal variables were assessed using the Fisher exact test. Continuous variables without normal distribution were stated as median and interquartile range (IQR), group differences were tested using the nonparametric Mann-Whitney U test. Kaplan-Meier curves were used to estimate the distribution of survival as a function of the follow-up duration. Optimal cut-off values were defined by Receiver operating characteristics (ROC) and Youden’s J statistic. The association of clinical, imaging and serological parameters with outcome was evaluated by uni- and multivariate Cox proportional-hazards regression models. Differences were considered statistically significant at *p* < 0.05.

## Results

Sixty-eight AL patients were included with a median follow up of 1.2 years (IQR, 0.3-4.1), of whom 44 (64.7%) did not survive this period. Patients’ characteristics are demonstrated in Table 1. Impairment of NYHA functional class (3 (IQR, 2-3) vs 2 (IQR, 2-3); *p* = 0.001) and Karnofsky index (85 ± 6 vs. 76 ± 10; *p* = 0.0001) were both accentuated in patients with primary endpoint. Regarding established serum biomarkers, only NT-proBNP showed significant differences between transplant-free survivors and patients with primary endpoint (1177 pg/mL (IQR, 528-3249) vs 3243 pg/mL (IQR, 1157-7505); *p* = 0.01). Among all annular locations, only AAPSE (8.8 mm (IQR, 6.9-10.4) vs 6.1 mm (IQR, 4.6-9.4); *p* = 0.02) and MAPSE_anterolateral_ (10.5 mm (IQR, 8.1-13.4) vs 7.3 mm (IQR, 5.4-11.7); *p* = 0.03) were significantly reduced in patients who died or were heart transplanted during the follow up period.

**Table 1:** Baseline Characteristics. Patients with primary combined endpoint versus transplant-free survivors (*n* = 68)

Parameter	Transplant-free Survivors (*n* = 24)	Heart transplantation or death (*n* = 44)	p
Age (yrs)	57 ± 8	59 ± 11	*n.s.*
Male (n)^a^	13 (54%)	27 (61%)	*n.s.*
BMI (kg/m^2^)	25 (23-27)	25 (23-27)	*n.s.*
Heart rate (bpm)	78 ± 13	80 ± 14	*n.s.*
NYHA class	2 (2-3)	3 (2-3)	*0.03*
Karnofsky Index	85 ± 6	76 ± 10	*0.0001*
*Laboratory data*			
MDRD (mL/min/1.73m^2^)	81 ± 17	83 ± 14	*n.s.*
NT-proBNP (pg/mL)	1177 (528-3249)	3243 (1157-7505)	*0.01*
cTNT ULN (n)^a^	8 (38%)	18 (41%)	*n.s.*
FLC difference (mg/dL)	1,1130 (435-5435)	3351 (975-8971)	*n.s.*
*CMR parameters*			
AAPSE (mm)	8.8 (6.9-10.4)	6.1 (4.6-9.4)	*0.02*
MAPSE anterior (mm)	8.1 (5.3-10.0)	6.2 (4.1-8.6)	*n.s.*
MAPSE anterolateral (mm)	10.5 (8.1-13.4)	7.3 (5.4-11.7)	*0.03*
MAPSE inferolateral (mm)	9.3 (6.3-12.1)	7.6 (5.2-12.2)	*n.s.*
MAPSE inferior (mm)	9.2 (7.4-11.1)	8.0 (5.0-11.7)	*n.s.*
MAPSE inferoseptal (mm)	7.6 (6.1-10.4)	7.1 (4.5-10.8)	*n.s.*
TAPSE (mm)	18.0 (10.0-22.4)	14.1 (9.0-19.7)	*n.s.*
GLS (%)	−14.8 ± 5.1	−13.2 ± 5.2	*n.s.*
LS_basal_ (%)	−12.3 ± 6.1	−11.4 ± 8.5	*n.s.*
LS_mid_ (%)	−16.4 ± 5.2	−14.7 ± 5.5	*n.s.*
LS_apical_ (%)	−17.6 ± 5.9	−16.2 ± 6.6	*n.s.*
Ejection Fraction (%)	62 ± 10	55 ± 13	*0.03*
Septal wall (mm)	14 ± 5	13 ± 3	*n.s.*
Lateral wall (mm)	10 ± 4	10 ± 4	*n.s.*
Relative LV Mass (g/m^2^)	75 ± 21	73 ± 35	*n.s.*
LA (mm)	39 ± 7	42 ± 7	*n.s.*
Relative LGE (%)	16.5 (10.0-35.7)	25.0 (18.1-30.4)	*n.s.*
Qualitative LGE (n)	2 (2-3)	2 (2-3)	*n.s.*

Normal distributed variables are presented as mean ± SD, non-parametric distributions are shown as Median (1st quartile-3rd quartile). ^a^dichotomous variable

*BMI* body mass index, *MDRD* glomerular filtration rate calculated with the formula of “Modification of Diet in Renal Disease”, *NT-proBNP* N-terminal prohormone of brain natriuretic peptide, *cTNT ULN* cardiac troponin T upper limit of normal, *FLC* free light chain difference, *AAPSE* anterior aortic plane systolic excursion, *MAPSE* mitral annular plane systolic excursion, *TAPSE* tricuspid annular plane systolic excursion, *GLS* global longitudinal strain, *LS*_*basal*_ mean longitudinal strain of basal segments 1-6, *LS*_*mid*_ mean longitudinal strain of midventricular segments 7-12, *LS*_*apical*_ mean longitudinal strain of apical segments 13-16, *LA* left atrium, *relative LGE* proportion of late gadolinium enhancement quantified by 5SD method, *qualitative LGE* Late Gadolinium Enhancement graduated in (1) none, (2) subendocardial or (3) transmural involvement

**Fig. 2:**
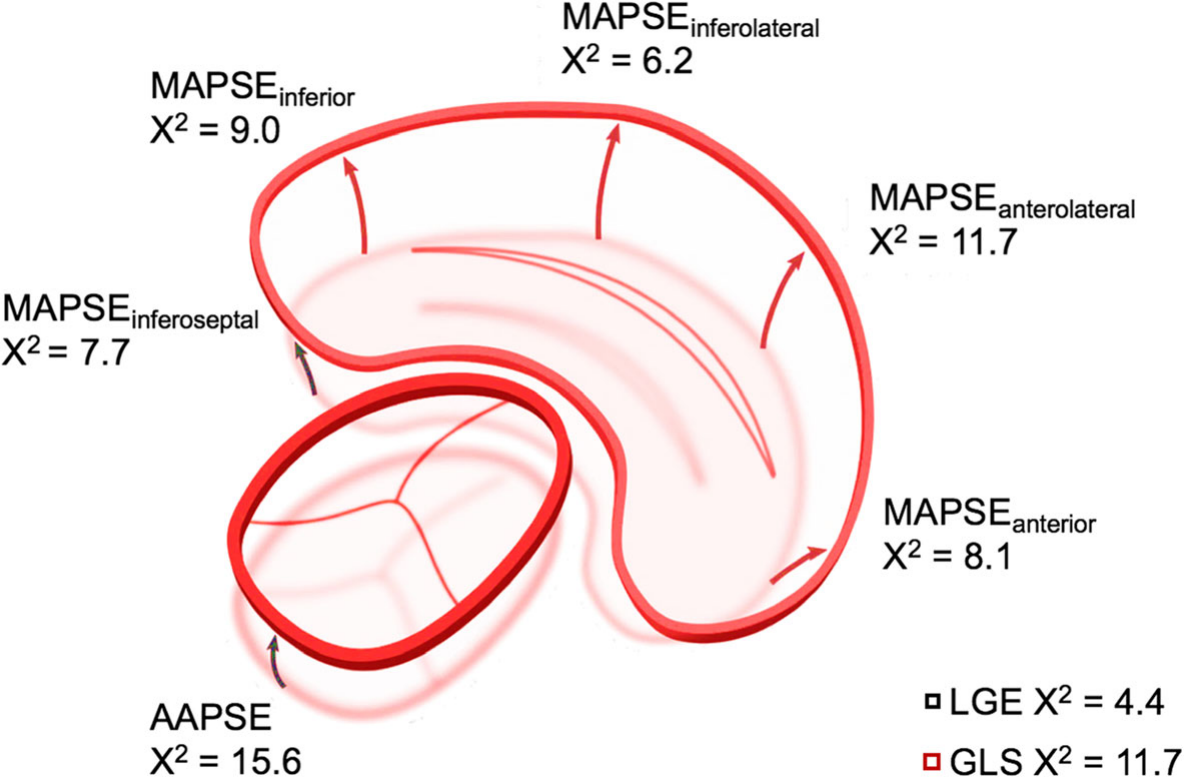
Distribution of the prognostic value for cardiac valve plane displacement on varying locations. Chi-square (X^2^) resulted from univariate regression analyses. To relate the prognostic value of cardiac valve plane motion with established predictors in AL amyloidosis relative LLGE and global longitudinal strain (GLS) are presented

AAPSE (χ^2^ = 15.6; *p* = 0.0002) was found the best predictor of transplant-free survival compared to all other valvular plane locations as well as relative/ qualitative LGE, GLS or regional longitudinal strain in univariate Cox-regression analysis (Fig. 2; Table 2). The slice-wise assessment of regional longitudinal strain did not reveal a significant benefit over GLS in terms of predictive power (Table 2). AAPSE (X^2^ = 21.6, *p* = 0.0001) was also found beneficial for predicting a combined endpoint of cardiac death, heart transplantation, and hospitalization compared to other established parameters (GLS: X^2^ = 11.4, *p* = 0.0007; relative LGE: X^2^ = 4.4, *p* = 0.03).

**Table 2:** Univariate Cox-regression for overall survival or heart transplantation (*n* = 68)

Variable	X^2^	HR	95% CI	p
NYHA class	**7.5**	1.75	1.15 to 2.68	*0.001*
Karnofsky Index	**21.3**	0.91	0.88 to 0.95	*0.0001*
*CMR parameters*				
AAPSE (mm)	**15.6**	0.10	0.03 to 0.34	*0.0002*
MAPSE anterior (mm)	**8.1**	0.19	0.06 to 0.60	*0.004*
MAPSE anterolateral (mm)	**11.7**	0.23	0.10 to 0.56	*0.001*
MAPSE inferolateral (mm)	**6.2**	0.36	0.16 to 0.83	*0.02*
MAPSE inferior (mm)	**9.0**	0.26	0.10 to 0.63	*0.003*
MAPSE inferoseptal (mm)	**7.7**	0.26	0.10 to 0.68	*0.01*
TAPSE (mm)	**6.7**	0.94	0.90 to 0.99	*0.01*
GLS (%)	**11.7**	1.12	1.05 to 1.19	*0.0006*
LS_basal_ (%)	**8.3**	1.08	1.02 to 1.13	*0.006*
LS_mid_ (%)	**12.8**	1.13	1.06 to 1.21	*0.0005*
LS_apical_ (%)	**6.9**	1.07	1.02 to 1.12	*0.005*
Ejection Fraction (%)	**11.6**	0.95	0.93 to 0.98	*0.0007*
Septal wall (mm)	**0.7**	1.03	0.96 to 1.11	*n.s.*
Relative LV Mass (g/m^2^)	**1.8**	1.01	0.99 to 1.02	*n.s.*
LA dilation ^a^	**0.02**	1.05	0.55 to 1.97	*n.s.*
Relative LGE (%)	**4.4**	1.02	1.00 to 1.04	*0.03*
Qualitative LGE (n)	**6.8**	1.50	1.09 to 2.06	*0.009*
*Laboratory data*				
cTNT ULN ^*a*^	**5.3**	1.94	1.09 to 3.45	*0.02*
log NT-proBNP (pg/mL)	**13.0**	1.52	1.20 to 1.94	*0.0006*
MDRD (mL/min/1.73m^2^)	**0.2**	1.00	0.99 to 1.01	*n.s.*
log FLC difference (mg/dL)	**6.3**	1.71	1.10 to 2.65	*0.02*

*X* ^*2*^ chi square, *HR* hazard ratio, *CI* confidence interval, ^a^dichotomous variable

*AAPSE* anterior aortic plane systolic excursion, *MAPSE* mitral annular plane systolic excursion, *TAPSE* tricuspid annular plane systolic excursion, *GLS* global longitudinal strain, *LS*_*basal*_ mean longitudinal strain of basal segments 1-6, *LS*_*mid*_ mean longitudinal strain of midventricular segments 7-12, *LS*_*apical*_ mean longitudinal strain of apical segments 13-16, *LA* left atrium, *relative LGE* proportion of late gadolinium enhancement quantified by 5SD method, *qualitative LGE* late gadolinium enhancement graduated in (1) none, (2) subendocardial or (3) transmural involvement, *cTNT ULN* cardiac troponin T upper limit of normal, *NT-proBNP* N-terminal prohormone of brain natriuretic peptide, *MDRD* glomerular filtration rate calculated with the formula of “Modification of Diet in Renal Disease”, *FLC* free light chain difference

**Fig. 3:**
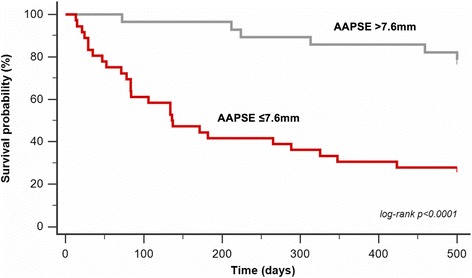
Kaplan-Meier survival curve demonstrates the discriminative prognostic value of AAPSE in cardiac biopsy proven AL amyloidosis. Cut-off (AAPSE ≤7.6 mm) was calculated by ROC analyses (AUC =0.80; CI: 0.68 to 0.89; *p* < 0.0001) and Youden’s-J statistic to predict all-cause death or heart transplantation within 6 months from index examination (*n* = 68)

An optimized cut-off value for AAPSE of ≤7.6 mm was calculated to predict whether patients would reach a primary endpoint within 6 months from index examination (AUC =0.80; CI: 0.68 to 0.89; *p* < 0.0001). The Kaplan-Meier survival curve is demonstrated in Fig. 3 (log-rank *p* ≤ 0.0001). Median survival of patients with AAPSE ≤7.6 mm was 0.5 years (IQR, 0.2-2.1) compared to 3.9 years (IQR, 1.5-5.8).

**Fig. 4:**
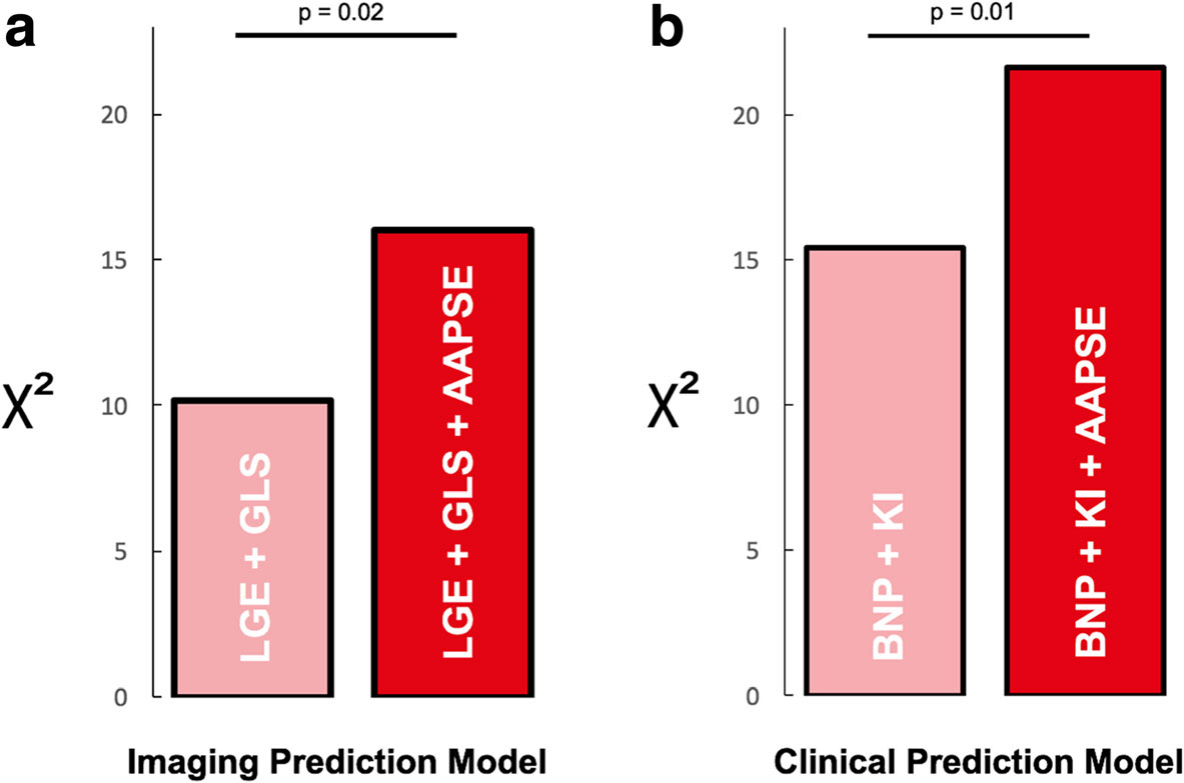
Incremental prognostic power of AAPSE. Sequential Cox-regression analysis was performed to evaluate the incremental predictive power of AAPSE for transplant-free survival in an (**a**) imaging (LGE and GLS; χ^2^ = 10.2) and a (**b**) clinical prediction model (NT-proBNP and Karnofsky Index; χ^2^ = 15.4). AAPSE significantly improved the predictive power of both models (imaging: AAPSE ∆χ^2^ = 5.8, *p* = 0.02; clinical: AAPSE ∆χ^2^ = 6.2, *p* = 0.01)

To further evaluate the incremental predictive power of AAPSE for transplant-free survival, a sequential Cox-regression was performed for a clinical (NT-proBNP and Karnofsky Index; χ^2^ = 15.4) and an imaging prediction model (LGE and GLS; χ^2^ = 10.2) (Fig. 4). Hereby, the stepwise addition of AAPSE provided a significant increase in predictive power for both models (clinical: AAPSE ∆χ^2^ = 6.2, *p* = 0.01; imaging: AAPSE ∆χ^2^ = 5.8, *p* = 0.02).

## Discussion

Our data indicate that in a head-to-head comparison in patients with biopsy-proven cardiac involvement in AL amyloidosis, the AAPSE appears to better predict outcome than measurements in other regions such as the mitral or tricuspid valve ring with incremental value to established clinical, biochemical, and imaging prognosticators.

The measurement of CVPD is traditionally reduced to MAPSE and TAPSE, which are both obtained in 4-chamber view on the lateral or septal mitral and on the lateral tricuspid annular location respectively [12]. However, recent findings have raised the hypothesis for significant heterogeneities of the prognostic value at different locations on the cardiac valve plane [14]. Therefore, the present data provides a comprehensive comparison and suggests the anterior aortic location (AAPSE) is associated with a better risk prediction in patients with AL amyloidosis. This finding cannot be easily explained, also because the motion of the aortic annulus is driven by a complex interplay between inserting myocardial fibers that are clockwise descending towards the apex and counteracting annular and aortic stiffness. Beyond risk prediction, future studies will have to address a potential role of AAPSE to indicate early myocardial manifestation or therapeutic benefits by guiding chemotherapeutic regimens in AL amyloidosis.

At present the most widely used staging system and validated gold standard to estimate the severity of cardiac impairment and life expectancy [17] is based on serum biomarkers NT-proBNP and cTNT [5], but serum levels depend on glomerular filtration rate, which is commonly affected in AL. Therefore, cardiac imaging parameters are gaining importance and our data provide further evidence that AAPSE may have an incremental predictive power to preexisting staging systems. As atrioventricular valve plane motion reflects the longitudinal shortening of the left ventricle, previous observations also support the significance of its impairment in AL. The relative longitudinal axes shortening (LAS) and global longitudinal strain (GLS) are both independent predictors of survival [6, 8]. Other innovative risk predictors in amyloidosis have emerged in cardiac magnetic resonance imaging, including late gadolinium enhancement, T1 mapping, and extracellular volume (ECV) which allow for a non-invasive semi-quantitative assessment of amyloid infiltration [18] and correlate with all-cause mortality [13, 19–21]. Recently, the prognostic value of left atrial function in AL was emphasized [22]. Nevertheless, there are obvious advantages of cardiac valve plane measurements over more refined imaging techniques for assessment of individual prognosis.

First, quantification is less time consuming than the post-processing and analysis of other approaches. Regarding particularly strain measurements, the presence of non-standardized tracking algorithms leads to variable results between different software solutions and insufficient tracking is a common source of errors. These issues have been demonstrated previously and are the main limitation for segmental analysis of longitudinal strain by feature tracking in CMR [23]. Moreover, the present data does not encourage for a routine assessment of slice-wise regional longitudinal strains as a significant benefit over GLS in terms of prognostication in AL amyloidosis could not be found. Second, measurements can be taken from conventional long axis views and don’t rely on additional sequences. Third, a non-contrast protocol avoids potential, albeit small risks associated with the administration of gadolinium chelates such as systemic nephrogenic fibrosis and gadolinium deposition in the human brain [24, 25]. Facing these issues, cardiac valve plain motion appears to allow a fast, cost-effective and reliable evaluation of prognosis. AAPSE may better predict mortality than global longitudinal strain according to the present analysis. This finding suggests, that differentiation of regional longitudinal function might be favorable over measurements of global longitudinal function for risk assessment. The typical pattern follows a gradient of impairment from basal to apical segments, which is usually termed as “apical sparing” [10, 11]. The fact that longitudinal shortening is the main contributor to left ventricular ejection fraction, might serve as an explanation why this contractile component is more sensitive to pathologic changes [9, 26], and eventually prognosis.

CVPD measurements were initially adopted from M-Mode echocardiography. Since then, several methods of measurement have been proposed [22, 27–30], while no standard technique has been recommended by official guidelines. The present analysis relies on a mode of measurement, that has previously demonstrated a good reproducibility between observers, studies, and modalities [15].

The present study has some limitations. 1) The data were obtained retrospectively and have to be confirmed in later, prospective samples. 2) The study was performed at a single-center and was restricted to patients with AL amyloidosis, which limits its generalizability. 3) The study cohort might be selection biased excluding patients unable to perform a CMR examination with rest dyspnea, severe arrhythmias, or claustrophobia.

## Conclusion

The predictive value of cardiac valve plane excursion varies between different annular locations. The Anterior Aortic Plane Systolic Excursion (AAPSE) appears to best predict all-cause mortality or heart transplantation in systemic AL amyloidosis with incremental prognostic value to established clinical, biochemical, and imaging prognosticators.

## Abbreviations

AAPSE: Anterior aortic plane systolic excursion
AL: Systemic light chain amyloidosis
BNP: Brain natriuretic peptide
bSSFP: Balanced steady state free precession
CMR: Cardiovascular magnetic resonance
CVPD: Cardiac valve plane displacement
GLS: Global longitudinal strain
IQR: Interquartile range
LGE: Late gadolinium enhancement
LV: Left ventricle/left ventricular
MAPSE: Mitral annular plane systolic excursion
TAPSE: Tricuspid annular plane systolic excursion.
